# Protective Ventilation During Controlled and Partial Ventilatory Support in ARDS: Clinical–Physiological Background and Monitoring

**DOI:** 10.3390/jcm15051830

**Published:** 2026-02-27

**Authors:** Rodrigo A. Cornejo, Caio C. A. Morais, Daniel H. Arellano, Roberto Brito, Abraham I. J. Gajardo, Marioli T. Lazo, Leonore B. D. Bos, Roberto González, Alejandro R. Bruhn, Jan Bakker

**Affiliations:** 1 Unidad de Pacientes Críticos, Departamento de Medicina, Hospital Clínico Universidad de Chile, Santiago 8380456, Chile; darellanos@uchile.cl (D.H.A.); roberto.britoa@gmail.com (R.B.); 2 Department of Intensive Care Adults, Erasmus MC University Medical Center, 3015 GD Rotterdam, The Netherlands; jan.bakker@erasmusmc.nl; 3 Unidade de Terapia Intensiva Adulto, Hospital das Clínicas Universidade Federal de Pernambuco, Recife 50670-901, Brazil; moraiscca@gmail.com; 4 Departamento de Kinesiología, Facultad de Medicina, Universidad de Chile, Santiago 8380453, Chile; 5 Keenan Research Centre, Li Ka Shing Knowledge Institute, St. Michael’s Hospital, Unity Health Toronto, Toronto, ON M5B 1W8, Canada; 6 Programa de Fisiopatología, Instituto de Ciencias Biomédicas, Facultad de Medicina, Universidad de Chile, Santiago 8380453, Chile; aij.gajardo@gmail.com; 7 Departamento de Kinesiología, Facultad de Medicina, Universidad Andrés Bello, Santiago 7591538, Chile; mariolilazocorona@gmail.com; 8 Erasmus MC University Medical Center, Faculty of Medicine, Erasmus University Rotterdam, 3015 GD Rotterdam, The Netherlands; louisebos24@gmail.com; 9 Departamento de Anestesia y Medicina Perioperatoria, Hospital Clínico Universidad de Chile, Santiago 8380456, Chile; rgonzalezcor@gmail.com; 10 Departamento de Medicina Intensiva, Facultad de Medicina, Pontificia Universidad Católica de Chile, Santiago 8331150, Chile; alejandrobruhn@gmail.com

**Keywords:** protective ventilation, acute respiratory distress syndrome, ventilator-induced lung injury, patient self-inflicted lung injury

## Abstract

Acute respiratory distress syndrome (ARDS) is characterized by severe hypoxemia, low lung compliance, and marked regional heterogeneity of aeration, making the lung highly vulnerable to injurious mechanical forces. Mechanical ventilation is essential to maintain gas exchange. However, excessive stress and strain may contribute to ventilator-induced lung injury (VILI). The progressive transition to partial ventilatory support introduces an additional risk: patient self-inflicted lung injury (P-SILI), driven by vigorous inspiratory efforts, large transpulmonary pressure swings, pendelluft, and heterogeneous regional strain. Advances in monitoring, imaging, and physiology-based management offer the potential to reduce lung injury and improve outcomes in mechanically ventilated patients with ARDS. This review aims to summarize the clinical–physiological background of VILI and P-SILI, describe protective strategies during controlled and partially assisted ventilation, and discuss monitoring tools to personalize mechanical ventilation in ARDS.

## 1. Introduction

Mechanical ventilation is a life-saving intervention in patients with acute respiratory distress syndrome (ARDS), yet it can paradoxically contribute to lung injury when applied to a structurally and functionally heterogeneous lung. Ventilator-induced lung injury (VILI) has long been recognized as a consequence of excessive mechanical forces, including overdistension, repetitive opening and closing of unstable lung units, and uneven distribution of stress and strain [1]. More recently, patient self-inflicted lung injury (P-SILI) has emerged as a complementary and clinically relevant concept, highlighting the potential for vigorous spontaneous breathing efforts to exacerbate lung damage through similar regional mechanical mechanisms [2]. Together, these phenomena underscore that lung injury during respiratory support is not solely determined by ventilator settings, but by the interaction between applied pressures, patient effort, and the underlying severity and distribution of lung disease.

Protective ventilation strategies have evolved in response to this understanding, moving beyond fixed ventilatory targets toward a more individualized, physiology-based approach. Limiting tidal volume and airway pressures remains foundational [3,4] with increasing attention directed toward driving pressure [5], mechanical power [6,7] and the regional distribution of ventilation [2]. Bedside monitoring of respiratory mechanics, along with emerging tools to assess lung heterogeneity, offers the potential to better align ventilatory support with the functional size and mechanical properties of the injured lung. This manuscript explores the mechanistic basis of VILI and P-SILI, examines current protective strategies, and discusses how physiological bedside monitoring may help mitigate lung injury while acknowledging the practical limitations and uncertainties that remain in clinical implementation.

This article is a narrative review and expert perspective based on the integration of the best available evidence with classical and contemporary physiological studies. The literature was identified through targeted searches of PubMed, Embase, and relevant bibliographies, focusing on experimental, translational, and clinical studies related to respiratory physiology and mechanical ventilation. Particular emphasis was placed on seminal physiological investigations, high-quality observational studies, randomized trials when available, and recent technological advances. Evidence was interpreted in light of established physiological principles and the authors’ own previously published work in this field. Given the perspective nature of this review, no formal systematic review methodology or predefined inclusion criteria were applied, and study selection was guided by relevance, methodological quality, and physiological plausibility.

## 2. Mechanical Ventilation and VILI in ARDS

ARDS is a complex clinical syndrome characterized by severe hypoxemic respiratory failure and reduced lung compliance due to rapidly developing lung inflammation and protein-rich, non-cardiogenic pulmonary edema resulting from severe alveolar–capillary barrier disruption and loss of normal aeration [8]. In ARDS, a substantial fraction of lung units becomes functionally unavailable because of consolidation, atelectasis or flooding, which produces functionally small lungs, condition termed “baby lung” [9]. In addition, marked regional heterogeneity of aeration is present, with non-aerated lung areas being more concentrated towards dependent and caudal regions [9].

Mechanical ventilation (MV) is the main supportive therapy for ARDS, leading to improvements in oxygenation and alveolar ventilation. However, due to the heterogeneity of the lung aeration that characterizes this syndrome, MV may also impose harmful increases in stress and strain on the remaining aerated lung tissue. This phenomenon is known as VILI [1].

Although direct measurement of stress and strain is not feasible in routine clinical practice, understanding their relationship is central for optimizing ventilator settings. Lung stress refers to the forces generated within the lungs in response to transpulmonary pressure (P_L_), defined as the pressure applied across the lung parenchyma [10]. Lung strain describes the lung deformation relative to resting lung volume and results from tidal inflation and positive end-expiratory pressure (PEEP). PEEP generates a baseline static strain that persists during expiration, onto which an additional dynamic strain is superimposed by tidal ventilation [10]. Experimental work by Protti et al. demonstrated that lung injury is primarily driven by cyclic strain, even when maximal strain levels are comparable [11].

Accordingly, the main mechanisms of VILI arise from excessive stress and strain within the lung parenchyma, leading to alveoli overstretch (overdistension), atelectrauma (repetitive alveolar opening and closing), barotrauma (rupture from high pressures), and secondary inflammatory responses known as biotrauma [1]. These mechanisms contribute to the progression of lung injury and may further aggravate the underlying disease process.

## 3. Spontaneous Breathing and P-SILI in ARDS

The transition from controlled to partial support ventilation is necessary to progress in the weaning process but is a highly vulnerable period for moderate–severe ARDS patients [12,13]. Superimposed spontaneous breathing (SB) on mechanical ventilation can occur without ventilatory support (unassisted SB) or be integrated in assisted ventilation modes (assisted SB). SB favors lighter sedation, as well as improvements in ventilation/perfusion matching dorsal lung ventilation, gas exchange, hemodynamics, and attenuate diaphragmatic atrophy, among other beneficial effects [14,15,16].

However, under certain conditions, SB has been demonstrated to cause or potentiate lung and respiratory muscle injury, a phenomenon termed P-SILI, which may complicate the weaning process [2,17]. This concept broadens the framework of VILI, integrating patient-generated forces into the pathophysiology of lung damage with important implications for clinical management.

P-SILI may occur when inspiratory effort becomes excessive. Several mechanisms have been proposed to elucidate how SB can lead to lung damage, including excessive intrathoracic pressure swing and high dynamic ΔP_L_, increased pulmonary transvascular pressure, expiratory muscle activity, and patient–ventilator asynchrony. In a large multicenter cohort, Dianti et al. [18] found that excessive respiratory effort was independently associated with increased ICU mortality and delayed liberation from mechanical ventilation, with high effort preceding overt increases in tidal volume (V_T_).

In addition, pendelluft represents a regional mechanism through which excessive inspiratory effort may contribute to P-SILI. Recently described in mechanically ventilated patients [2], pendelluft is characterized by an inefficient and potentially injurious pattern of lung inflation, involving the intrapulmonary redistribution of air from non-dependent to dependent lung regions during the early stage of inspiration, without a significant change in tidal volume. Pendelluft has been demonstrated at the bedside using electrical impedance tomography (EIT) and is primarily driven by localized negative pleural pressure generated by diaphragmatic contraction. Importantly, pendelluft is directly associated with tidal recruitment, regional lung overstretch, and increased regional transpulmonary pressure [2]. As such, it provides a clinically relevant surrogate of regional lung injury mechanisms and can be continuously monitored non-invasively at the bedside.

## 4. Similarities Between VILI and P-SILI

VILI and P-SILI share certain physiological determinants at the regional level that likely activate similar inflammatory pathways [2,19,20,21]. In ARDS patients under controlled MV, dynamic strain has been shown to correlate with interleukin-6 (IL-6) and interleukin-8 (IL-8) concentrations in bronchoalveolar lavage fluid [20]. Likewise, metabolic activity in normally aerated lung regions correlates with plateau pressure, a surrogate of stress, and with tidal volume (V_T_) normalized by end-expiratory gas volume, reflecting strain [21].

During spontaneously breathing, experimental studies suggest that intense respiratory effort induces inflammation predominantly in dependent lung regions, as evidenced by PET imaging and histological findings [22,23]. Clinically, high respiratory drive and high pendelluft magnitude have been linked to elevated levels of IL-8, IL-18, and Caspase-1 at the onset of spontaneous breathing in ARDS patients [24,25]. Biotrauma, a mechanism implicated in VILI (and potentially P-SILI) appears to be mediated by mechanotransduction in alveolar cells and macrophages via reactive oxygen species production, involving several molecular signaling pathways, including activation of the NLRP3 inflammasome [1,26,27]. Notably, biotrauma exerts effects not only locally but also systemically, contributing to distant organ dysfunction [1,28].

## 5. Current Protective Strategies to Reduce the Risk of VILI

VILI may be an important cause of poor clinical outcomes in ARDS patients [3]. Therefore, different strategies aiming to reduce the incidence and severity of VILI have been introduced, such as low V_T_ ventilation, prone positioning, extracorporeal membrane oxygenation (ECMO) and the use of neuromuscular blocking agents [3,29,30,31].

The ARDS Network ARMA trial demonstrated that a strategy using a of V_T_ ~6 mL/kg of predicted body weight (PBW) and a plateau pressure lower than 30 cm H_2_O, compared with the traditional V_T_ of ~12 mL/kg, significantly reduced mortality in ARDS patients [3]. However, the lung-protective benefit of a V_T_ ~6 mL/kg of PBW appears to be effective only when accompanied by a safe driving pressure (≤15 cm H_2_O), thereby limiting dynamic stress and strain [5]. The use of normalized elastance, defined as the ratio between driving pressure and V_T_ in mL/kg PBW, may further facilitate the implementation of truly protective ventilation [32]. Nonetheless, reliance on standard PBW formulas to prescribe V_T_ often results in exceeding a patient’s actual pre-injury ‘safe’ tidal lung volume in individuals who are older, shorter, female, or from non-White populations [33].

Prone positioning improves oxygenation and, by recruiting non-aerated tissue and by reducing the vertical pleural pressure gradient, may provide a more uniform distribution of P_L_, decreasing lung stress and strain in the ARDS lung [34,35]. In addition, prone positioning may enhance the beneficial effects of high PEEP on lung recruitment and reduction in tidal recruitment and preventing the negative impact of PEEP on tidal hyperinflation, compared to supine position [35]. Nevertheless, the level of PEEP should be titrated during prone [35,36,37]. Prone positioning is typically delivered in daily sessions of 16–18 h, separated by variable periods spent in the supine position that may last several hours [29]. Many patients require repeated sessions to achieve and maintain adequate gas exchange. However, these daily transitions pose several challenges, including increased staff workload, the risk of oxygenation deterioration, adverse events associated with repositioning, and interruptions in lung-protective ventilation. To mitigate these drawbacks, extended prone sessions lasting more than 24 h have been adopted in some centers [38,39]. This prolonged strategy may reduce the disadvantages inherent to daily cycling and offers operational benefits. Notably, a retrospective study reported a potential mortality reduction with extended sessions [40], although prospective studies are needed to confirm these observations.

PEEP promotes alveolar recruitment and prevents cyclic derecruitment, attenuates end-expiratory mechanical stress, and facilitates the delivery of adequate and more homogeneously distributed tidal ventilation. However, inappropriate high PEEP levels can increase static stress, impact hemodynamics, and worsen clinical outcomes [41]. Therefore, individualized PEEP levels, determined through methods like esophageal manometry [42] or EIT, are recommended to achieve optimal effectiveness. Recently, an EIT-guided PEEP strategy, based on the crossing point between overdistension and collapse during a decremental PEEP trial, was compared with a conventional lower PEEP/FiO_2_ table in patients with moderate-to-severe ARDS. The EIT-guided approach did not improve overall survival. However, in a prespecified subgroup of patients with higher lung recruitability, EIT-guided PEEP was associated with lower mortality. In contrast, no benefit was observed in patients with low recruitability, with a non-significant trend toward worse outcomes in the EIT group despite similar PEEP levels compared to control group [43]. Therefore, assessment of lung recruitability—such as potentially recruitable lung or the recruitment-to-inflation ratio [44,45], as applied in the EITVent trial by Yuan et al. [43], may help identify patients who are more likely to benefit from higher PEEP levels. In addition, the ARDS lung morphology (focal versus non-focal) might guide PEEP titration, with higher PEEP levels being more appropriate in patients with non-focal ARDS [46]. However, substantial interindividual variability in patient responses has been observed, and there is currently a lack of definitive clinical trials supporting specific methods.

ECMO is a potentially lifesaving strategy in the most severe form of ARDS. A recent systematic review and individual patient data meta-analysis showed that 90-day mortality was significantly lowered by ECMO compared with conventional management [30]. ECMO is an option for patients with severe respiratory failure who do not respond to conventional therapies, which allows for ultra-lung-protective ventilation by providing extracorporeal gas exchange, thereby reducing the mechanical forces applied to the lungs [47]. During the H1N1 pandemic and, more recently, throughout the COVID-19 pandemic, ECMO played an important role, allowing the most severely ill patients to be supported with more than reasonable outcomes. The implementation of a rationalization process, defined by the establishment of tertiary referral centers, peer-based patient selection, a transportation program for ECMO candidates (or mobile ECMO), strict follow-up protocols, and public–private cooperation, can ensure cost-effective ECMO allocation and potentially improve patient outcomes in a resource-limited and logistically complex pandemic scenario [48].

High-frequency oscillatory ventilation (HFOV) has been used as a rescue strategy for refractory hypoxemia in severe ARDS, rather than as a first-line ventilation mode. By delivering very small tidal volumes around a constant mean airway pressure at high oscillatory frequencies, HFOV aims to limit volutrauma and atelectrauma by reducing cyclic alveolar collapse and overdistension. However, HFOV is associated with significant hemodynamic effects, particularly impaired venous return and reduced cardiac output. Large randomized clinical trials have failed to demonstrate a survival benefit and, in some cases, have shown potential harm [49,50]. Consequently, routine use of HFOV in adult ARDS is not recommended.

## 6. Current Protective Strategies to Reduce the Risk of P-SILI

Enabling SB with an optimal level of inspiratory effort is essential for patients with acute respiratory failure on mechanical ventilation. Inadequate or absent effort can lead to diaphragmatic dysfunction, complicating weaning and increasing the risk of atelectasis and hypoxemia [51,52]. Conversely, vigorous effort can generate excessive negative alveolar pressures, heightening the risk of P-SILI [2,17]. Different modifiers of inspiratory effort and respiratory drive, such as low-pressure support, inspiratory sub-assistance, external resistances and lower PEEP levels, can favor P-SILI and myotrauma [2,52,53].

The recommendation is to transition to a partial support ventilation mode once the patient’s PaO_2_/FiO_2_ ratio improves (≥150 mmHg) and arterial pH exceeds 7.35. Daily evaluations are advised to assess readiness for this switch. Upon transition, monitoring respiratory drive and effort is recommended.

Pressure-controlled strategy allowing non-synchronized unassisted spontaneous ventilation (PC-SV) or airway pressure realize ventilation (APRV) have demonstrated to generate lower V_T_ and P_L_ than fully synchronized or partially synchronized pressure-targeted modes despite similar ventilatory settings [54]. PC-SV has been associated with a reduction in the need for sedatives and the use of alternative therapies for managing hypoxemia [55]. However, PC-SV is not free from pendelluft [25]. This could explain the lack of impact of this ventilatory strategy in major outcomes [55]. Closed-loop ventilation modes, including Adaptive Support Ventilation (ASV), INTELLiVENT-ASV (Hamilton Medical AG, Rhäzüns, Switzerland), Neurally Adjusted Ventilatory Assist (NAVA, Maquet, Solna, Sweden), and Proportional Assist Ventilation Plus (PAV+, Medtronic, Minneapolis, MN, USA), may facilitate lung-protective ventilation and improve patient–ventilator synchrony by automatically adjusting tidal volume and respiratory rate in response to patient-specific respiratory mechanics and drive [56,57]. However, these modes do not directly quantify lung stress or strain, nor do they assess regional ventilation or lung heterogeneity. Accordingly, they should be viewed as supportive strategies rather than substitutes for individualized clinical assessment and decision-making in patients with ARDS.

The transition to PSV should be guided by routine clinical parameters, with the goals of maintaining patient comfort and ensuring adequate ventilation and oxygenation. The application of a systematic protocol was recently incorporated in a randomized controlled trial (RCT). During PSV, target values include a respiratory rate (RR) of 12–35 breaths/min, VT of 5–10 mL/kg PBW, arterial pH between 7.32 and 7.47, and arterial oxygen saturation (SpO_2_) of 90–96%, using the lowest FiO_2_ necessary to achieve these targets [58]. The monitoring of these parameters will facilitate early identification of respiratory distress or acid–base disturbances. Patients should be reassessed immediately after PSV initiation and at frequent intervals thereafter.

PSV is initiated with a PS level of 10–20 cm H_2_O (or the level used prior to transition), while maintaining the previous PEEP and FiO_2_ settings. Appropriate trigger sensitivity and alarm limits should be set to ensure patient safety. Ventilator adjustments are guided by ongoing clinical assessment and gas exchange, with particular attention to the presence or absence of respiratory distress. Respiratory distress has been defined by the presence of at least two clinical signs, including hypoxemia, sustained tachypnea, hemodynamic instability, marked dyspnea, anxiety, or use of accessory respiratory muscles, consistent with prior definitions [58]. When respiratory distress is present, potentially reversible causes—such as airway secretions, bronchospasm, pulmonary edema, or metabolic acidosis—should be promptly identified and treated. PS should then be increased in small increments until respiratory distress resolves and arterial pH exceeds 7.32. PEEP and/or FiO_2_ should be adjusted as needed to maintain adequate oxygenation. In the absence of respiratory distress, PEEP and FiO_2_ should be adjusted according to clinical judgment, targeting the lowest FiO_2_ required to maintain SpO_2_ between 90% and 96%. If RR and VT remain within target ranges, no ventilator adjustments are required. If ventilation deviates from these targets, arterial blood gas analysis should guide further management: respiratory alkalemia should prompt evaluation and correction of causes of hyperventilation, followed by stepwise reduction in pressure support, whereas respiratory acidemia or the emergence of respiratory distress should prompt incremental increases in pressure support, with conversion to assist/control ventilation if safety limits are reached [58].

In an effort to meet lung and diaphragm protective targets at the SB onset, Dianti J. et al. developed a physiological trial in patients with moderate to severe acute hypoxemic respiratory failure integrating different strategies [59]. The targets were safely and effectively achieved by systematically titrating support ventilation and sedation, adjusting PEEP, increasing sweep gas flow (in patients on VV-ECMO), and, if needed, administering partial neuromuscular blockade. However, the impact of this approach in clinical outcomes needs to be further assessed. A recent study assessed if a ventilatory strategy that allows early SB, but limits total driving pressure until extubation, could be superior to a standard low tidal volume ventilation. The extended lung protection strategy included a stepwise protocol guided by EIT aimed at minimizing driving pressure and respiratory drive. The authors found a better oxygenation and sustained improvement of X-ray and compliance, and a shorter time from randomization to room-air breathing in the intervention group, but no difference in primary outcome (the modified lung injury score) or mortality were observed [60].

Higher levels of PEEP and pressure support (PS) may help reduce inspiratory effort and minimize the magnitude of pendelluft [61,62,63]. However, their physiological effects are variable and appear to be influenced by patient-specific factors. For example, PEEP has been shown to reduce esophageal pressure swings, particularly when it leads to improved respiratory system compliance and greater patient comfort [59]. Likewise, decreasing PS can increase pendelluft, especially in patients with initially high pendelluft levels [62]. Importantly, only an optimal PEEP promotes a more uniform distribution of negative pleural pressure swings, which may reduce inspiratory dyssynchrony and attenuate regional lung injury, especially in dependent lung regions [22]. Nevertheless, a higher PEEP can also influence the diaphragm’s force-generating capacity during SB [64] and has been associated with an increase in expiratory activity, which may decrease the efficiency of PEEP in reducing pendelluft [61].

Prone positioning, regardless of PEEP levels, may reduce the uneven distribution of lung stress and inflation caused by spontaneous breathing efforts, and attenuate inspiratory effort, thereby decreasing the risk of effort-dependent lung injury [64]. Additionally, prone positioning may reduce the magnitude of pendelluft and improve neuromuscular coupling [65].

Patients should be transitioned back to A/C ventilation if safety thresholds are exceeded, such as high pressure support (>20 cm H_2_O), high FiO_2_ (>60%), excessive airway pressures (PS plus PEEP > 30 cm H_2_O), the development of clinical instability, or inability to trigger the ventilator due to the requirement for deep sedation and/or neuromuscular blockade. Patients who require a return to A/C ventilation should be reassessed within 24 h, and at least daily thereafter, for readiness to resume PSV [58].

## 7. Operational Bedside Monitoring to Detect VILI and P-SILI Risk

Given the shared mechanistic pathways underlying VILI and P-SILI, bedside monitoring should focus on identifying excessive lung-distending pressures and alveoli deformation using tools that are readily available and non-invasive. This approach is particularly relevant during transitions from controlled to assisted ventilation, when patient-generated pressures may substantially contribute to lung stress. In this context, the optimal monitoring approach should consider the clinical phase, the variables of interest, and the thresholds for clinical intervention. Key non-invasive monitoring variables, their methods of assessment, reference ranges, and clinical implications are summarized in Table 1.

**Figure 1:**
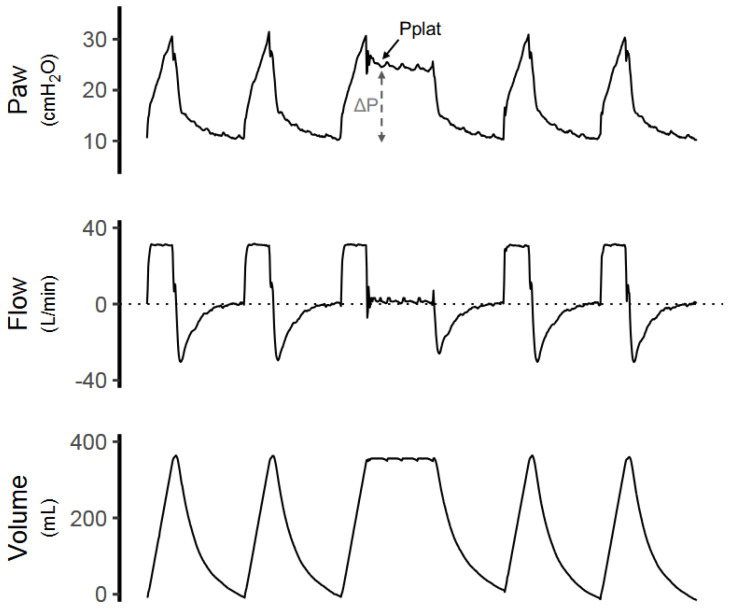
**Measurement of Plateau Pressure and Driving Pressure During Controlled Mechanical Ventilation.** Representative airway pressure (Paw), flow, and volume waveforms obtained during controlled mechanical ventilation are shown. An inspiratory hold maneuver is performed at end-inspiration, resulting in a period of zero flow. Plateau pressure (Pplat) is measured at the earliest point after inspiratory flow reaches zero. Driving pressure (ΔP) is calculated as the difference between Pplat and positive end-expiratory pressure (PEEP). Measuring Pplat immediately at the cessation of flow minimizes underestimation of driving pressure that may occur due to time-dependent stress relaxation of the respiratory system during prolonged inspiratory holds.

During controlled mechanical ventilation, the risk of VILI is primarily assessed using ventilator-derived parameters reflecting global lung stress. Driving pressure (ΔP = Pplat − PEEP) should ideally be maintained ≤15 cm H_2_O (Figure 1), as higher values have been consistently associated with increased mortality [5]. Plateau pressure should generally remain ≤30 cm H_2_O [3]. These variables provide pragmatic, non-invasive surrogates of dynamic lung strain and remain central targets for lung-protective ventilation.

**Figure 2:**
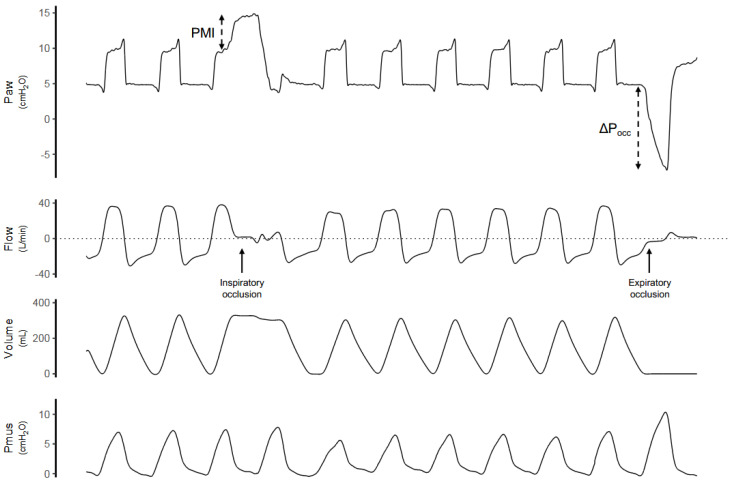
**Measurement of Plateau Pressure, Muscle Pressure Index, and Occlusion Pressure During Assisted Ventilation**. Representative airway pressure (Paw), flow, volume, and estimated respiratory muscle pressure (Pmus) waveforms obtained in a patient receiving pressure support ventilation (PSV) are shown. An inspiratory occlusion maneuver is performed during assisted ventilation, resulting in a brief period of zero flow, which allows measurement of plateau pressure and estimation of the pressure–muscle index (PMI) from the airway pressure waveform during the inspiratory pause. An expiratory occlusion maneuver is subsequently applied at end-expiration, and the resulting negative deflection in airway pressure is used to calculate the occlusion pressure (ΔPocc), an index of inspiratory effort. Together, inspiratory and expiratory occlusions enable assessment of respiratory system mechanics and patient inspiratory drive during assisted ventilation.

During assisted ventilation, assessment of lung injury risk must incorporate patient-generated forces, which can be estimated non-invasively [66]. Plateau and driving pressures can also be assessed during assisted ventilation by performing end-inspiratory occlusion maneuvers in spontaneous breathing modes (Figure 2). Under these conditions, a plateau pressure higher than peak airway pressure reflects the combined contributions of ventilator assistance and inspiratory muscle effort [66]. The resulting driving pressure has been independently associated with increased mortality in patients with ARDS receiving assisted mechanical ventilation [67]. The difference between plateau and peak airway pressure measured during end-inspiratory occlusion represents the pressure generated by the patient’s inspiratory muscles and corresponds to the muscular pressure index (PMI). PMI, therefore, provides a non-invasive estimate of inspiratory effort and may be used to guide ventilator adjustments, such as the level of pressure support [68].

Inspiratory effort can also be monitored using Pocc [66,69], measured as the negative pressure deflection during a brief end-expiratory occlusion (Figure 2). Pocc provides an estimate of inspiratory muscle pressure (Pmus ≈ −0.75 × Pocc) and correlates with esophageal pressure swings without requiring invasive monitoring. Prospective data demonstrate a non-linear association between Pocc and clinical outcomes, with both insufficient and excessive effort associated with worse outcomes. In patients with more severe hypoxemia, intermediate levels of inspiratory effort, corresponding to Pocc values approximately between −10 and −20 cm H_2_O, were associated with lower mortality and higher rates of ICU discharge, whereas values outside this range were associated with worse outcomes [18].

Pocc also enables estimation of dynamic transpulmonary driving pressure (ΔPL,dyn ≈ ΔPaw,dyn − 0.66 × Pocc), which reflects the total lung-distending pressure resulting from the combined effects of ventilator insufflation and patient effort. Persistently elevated ΔPL,dyn values (>16 cm H_2_O) should raise concern for injurious lung stress, regardless of whether the predominant contributor is ventilator-delivered pressure or spontaneous effort [66,69]. Elevated ΔPL,dyn has been independently associated with increased mortality and prolonged ICU stay, supporting its clinical relevance as a non-invasive integrative marker of VILI and P-SILI risk.

Respiratory drive reflects the intensity of output generated by the respiratory centers. The decrease in airway pressure during the first 100 milliseconds of an end-expiratory occlusion or P0.1, provides a non-invasive estimate of respiratory drive. A P0.1 value between 1.0 and 4.0 cm H_2_O is generally considered within the normal range [66]. Importantly, both abnormally low and excessively high values of P0.1 have also been associated with increased ICU mortality and lower rates of ICU discharge, particularly among patients with a PaO_2_/FiO_2_ ratio below 150 mmHg [18]. Importantly, there are no universally accepted or validated threshold values for inspiratory effort variables; consequently, their interpretation should be individualized and integrated with clinical assessment.

**Figure 3:**
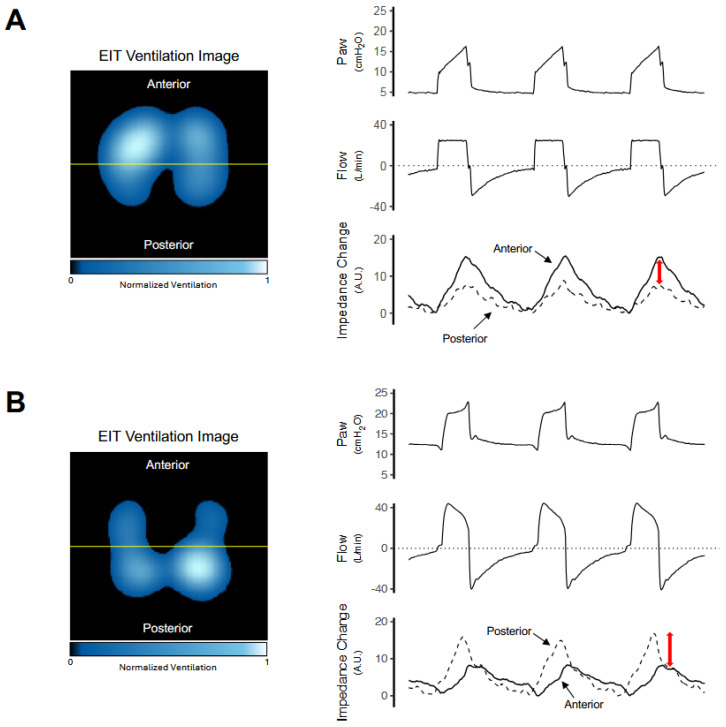
**Regional ventilation distribution during controlled mechanical ventilation and spontaneous breathing assessed by electrical impedance tomography**. Panel (**A**) illustrates a patient under controlled mechanical ventilation. Electrical impedance tomography (EIT) ventilation imaging demonstrates heterogeneous ventilation distribution with predominance in the anterior lung regions. Corresponding time-aligned waveforms of airway pressure (Paw), flow, and regional impedance change show that the anterior region (solid line) exhibits approximately twice the tidal impedance variation compared with the posterior region (dashed line), indicating marked regional disparity in tidal ventilation during controlled ventilation (red arrow). Panel (**B**) shows an example during spontaneous breathing. EIT imaging again reveals heterogeneous ventilation distribution, but with a shift toward greater ventilation in the posterior lung regions. This redistribution is more clearly reflected in the impedance waveforms, in which the posterior region (dashed line) demonstrates nearly double the regional impedance change compared with the anterior region (red arrow). Asynchronous impedance changes between regions are observed, consistent with intrapulmonary gas redistribution (pendelluft). In both conditions, pronounced regional ventilation heterogeneity is evident, which may be associated with uneven regional lung stress and strain.

Finally, regional non-invasive monitoring techniques [70], such as electrical impedance tomography (EIT), provide complementary information by detecting heterogeneous ventilation during controlled ventilation (Figure 3A) and regional phenomena during spontaneous breathing, such as pendelluft, which may occur despite apparently protective global ventilatory parameters (Figure 3B; see figure legend for details) [2]. In addition to practical issues such as electrode placement, signal quality, and data interpretation, EIT assesses only a single transverse lung slice; therefore, craniocaudal heterogeneity—particularly at trunk elevations greater than 30°—may limit its representativeness. EIT-derived pendelluft depends on the analytical method used, and no validated cutoff exists to define clinically relevant pendelluft magnitude. As EIT does not directly measure airflow, pendelluft is inferred from regional impedance changes and is influenced by limited spatial resolution and assumptions of a linear impedance–volume relationship. Phase-based methods are noise-sensitive and non-quantitative, intra-tidal redistribution approaches may be confounded by cyclic recruitment or overdistension, and flow- or pressure-referenced methods require precise synchronization and are susceptible to leaks and irregular breathing. Quantitative estimates therefore provide only relative measures. Nevertheless, EIT offers unique regional information not captured by global respiratory mechanics alone. Notably, detection of EIT-derived pendelluft in mechanically ventilated patients may support optimization of ventilator settings and potentially improve outcomes in spontaneously breathing patient [71]. Further studies are needed to confirm these findings, and standardized strategies for integrating EIT into ventilator management are still lacking. Integrating global ventilatory variables, non-invasive measures of effort and lung stress, and regional imaging allows a more comprehensive and individualized assessment of lung injury risk at the bedside.

## 8. Personalized Mechanical Ventilation and Future Directions

These monitoring strategies provide a holistic approach to reducing the risk of lung injury, balancing effective oxygenation and ventilation, while minimizing lung stress and strain, preserving lung integrity, and improving outcomes for ventilated patients. Modern approaches emphasize personalized mechanical ventilation, incorporating both static parameters (e.g., V_T_, PEEP, plateau pressure, and P_L_) and dynamic parameters (e.g., respiratory rate and airflow patterns). Recent innovations, such as mechanical power [6] (quantifying lung energy transfer per cycle) and a novel index [7] ([4 × Driving Pressure] + Respiratory Rate), underscore the role of ventilation intensity in VILI risk. These advancements represent a shift toward more nuanced and individualized ventilation strategies. Future directions in the management of VILI and P-SILI include the development of new ventilatory modes that adapt to patient-specific lung mechanics, the use of biomarkers to predict and monitor lung and diaphragm injury, and the integration of artificial intelligence to optimize ventilatory settings in real-time according to specific ARDS subphenotypes. The integration of precision medicine into mechanical ventilation represents a promising avenue for improving outcomes in ARDS patients.

## 9. Conclusions

Protective ventilation is an evolving concept. Recent studies have clarified that protective ventilation must extend beyond static ventilator settings to encompass the interaction between ventilator-delivered pressure and patient-generated effort. Avoiding VILI and P-SILI remains a critical goal in the management of patients requiring respiratory support. Advances in monitoring and management strategies have contributed to a better understanding and reduction in these injuries. Individualized ventilation strategies, coupled with adjunctive therapies and emerging technologies, offer the potential to further mitigate the risks associated with mechanical ventilation. As research continues to evolve, the focus will be on integrating these advancements into clinical practice to improve patient outcomes.

## Figures and Tables

**Table 1:** Operational bedside monitoring variables to detect VILI and P-SILI risk in Mechanically Ventilated Patients.

Domain	Monitoring Variable	Definition/Measurement	Clinical Role (Why It Matters)	Recommended Targets */ Interpretation
Ventilatory Mechanics	Tidal Volume (VT)	Volume delivered per breath (mL/kg PBW).	High VT increases dynamic strain and VILI risk, especially in ARDS with reduced functional lung size (“baby lung”).	4–8 mL/kg PBW for lung-protective ventilation.
	Plateau Pressure (Pplat)	Measured by end-inspiratory occlusion during passive or assisted ventilation. Reflects static distending pressure of the respiratory system.	Surrogate for alveolar pressure; elevated values increase barotrauma and volutrauma risk.	<30 cm H_2_O recommended. Higher values reflect reduced compliance.
	Driving Pressure (ΔP)	Difference between Pplat and PEEP.	Strong independent predictor of mortality in ARDS; reflects lung size available for ventilation (controlled and assisted ventilation).	<15 cm H_2_O preferred.
	Respiratory System Compliance (CRS)	VT/ΔP.	Declines with ARDS severity; reflects “baby lung.” Longitudinal trends indicate improvement or deterioration.	Reduced CRS = higher VILI susceptibility.
	Transpulmonary Pressure (PL = Paw − Pes)	True distending pressure of the lung.	Guides individualized PEEP; avoids overdistension while preventing collapse.	In passive ARDS: Pes-guided PEEP improves oxygenation and may reduce VILI.
Work and Effort of Breathing	Pmus (Muscle Pressure)	Pressure generated by respiratory muscles: Pmus = Pes − chest wall recoil pressure.	Differentiates patient effort from ventilator support; high Pmus = risk of P-SILI.	Peak Pmus ≥ 5 and <15 cm H_2_O recommended.
	Pressure Muscle Index (PMI)	Index obtained during PSV when an end-inspiratory pause reveals a difference between peak and plateau airway pressure	Estimate of inspiratory muscle effort during assisted ventilation. High PMI increases P-SILI risk, diaphragmatic overload. Useful for adjusting the assistance during PSV	PMI > 0 and <6 cm H_2_O considered safe.
	Pocc (Airway Occlusion Pressure Drop)	Drop in Paw during a full inspiratory occlusion.	Non-invasive surrogate of ΔPes and Pmus during tidal breathing.	Pmus ≈ Pocc × (−0.75); ΔPes ≈ Pocc × (−0.66).
Respiratory Drive	P 0.1	Airway pressure drop in first 100 ms of an occluded breath.	Reflects neural respiratory drive; independent of effort/performance capacity.	1–4 cm H_2_O normal range. Values > 5 = high drive.
Regional Lung Imaging & Monitoring	Electrical Impedance Tomography (EIT)	Bedside monitoring using thoracic electrode belt to estimate regional lung aeration and ventilation distribution.	Real-time assessment of overdistension, collapse, pendelluft, and dynamic strain; guides PEEP titration and synchrony.	Detects: regional ventilation, global inhomogeneity, ΔZ, pendelluft, regional strain
	Pendelluft (via EIT)	Intratidal gas redistribution between lung regions due to asynchronous filling.	Marker of heterogeneous effort, regional stress/strain, risk of P-SILI during spontaneous breathing.	Large pendelluft signals suggest need for unloading, sedation or PEEP adjusting

* There are no universally validated “safe” cut-offs for these parameters. The ranges proposed in this Table have been suggested in the literature and expert practice as reasonable targets.

## Data Availability

No new data were created or analyzed in this study. Data sharing is not applicable to this article.
